# Medial‐pivot design total knee arthroplasty provides higher post‐operative patient satisfaction than posterior‐stabilized design under mechanical alignment

**DOI:** 10.1002/ksa.70111

**Published:** 2025-10-27

**Authors:** Sho Masuda, Yukihide Minoda, Hideki Ueyama, Yohei Ohyama, Ryo Sugama, Hidetomi Terai

**Affiliations:** ^1^ Department of Orthopaedic Surgery Osaka Metropolitan University Graduate School of Medicine Osaka Japan; ^2^ Department of Orthopaedic Surgery Osaka Rosai Hospital Sakai Japan

**Keywords:** mechanical alignment, medial‐pivot, patient‐reported outcome measures, posterior‐stabilized, total knee arthroplasty

## Abstract

**Purpose:**

Numerous studies have compared post‐operative patient‐reported outcome measures (PROMs) between medial‐pivot design total knee arthroplasty (MP‐TKA) and posterior‐stabilized design TKA (PS‐TKA); however, whether MP‐TKA demonstrates superior outcomes remains controversial. This study aimed to compare post‐operative PROMs between MP‐TKA and PS‐TKA under mechanical alignment, using implants from the same manufacturer to eliminate the influence of shape, thereby focusing solely on the differences in articular surface design.

**Methods:**

A total of 141 TKAs from the same manufacturer were retrospectively assessed, comprising 71 MP‐TKAs and 70 PS‐TKAs. Knee range of motion and PROMs, including the Knee Society Score‐2011 (KSS‐2011) and Forgotten Joint Score‐12 (FJS‐12), were evaluated at 3, 6 and 12 months post‐operatively and annually thereafter, and compared between the MP‐TKA and PS‐TKA groups. The mean follow‐up period was 51 ± 13 and 50 ± 17 months in the MP‐TKA and PS‐TKA groups, respectively (*p* = 0.535).

**Results:**

The post‐operative knee flexion angle was smaller in the MP‐TKA group than in the PS‐TKA group (124 ± 13 vs. 132 ± 13, *p* < 0.001). However, the change in knee flexion angle from the preoperative values between the two groups (5 ± 15 vs. 6 ± 15, *p* = 0.752) showed no difference. Compared with the PS‐TKA group, the MP‐TKA group showed higher KSS‐2011 scores for symptoms (22 ± 3 vs. 19 ± 6, *p* = 0.002), satisfaction (32 ± 8 vs. 28 ± 10, *p* = 0.020) and functional activities (79 ± 16 vs. 71 ± 22, *p* = 0.041). The MP‐TKA group had a higher FJS‐12 total score than did the PS‐TKA group (73 ± 24 vs. 64 ± 27, *p* = 0.025).

**Conclusions:**

MP‐TKA demonstrated better PROMs than did PS‐TKA under mechanical alignment. Implant design with greater antero‐posterior stability could improve post‐operative outcomes after TKA.

**Level of Evidence:**

Level III.

**Abbreviations**
3D: three‐dimensional
aHKA: arithmetic hip–knee–ankle angle
BMI: body mass index
CPAK: coronal plane alignment of the knee
CT: computed tomography
FJS: Forgotten Joint Score
FMA: femoral mechanical angle
HKA: hip–knee–ankle angle
KSS: Knee Society Score
LDFA: lateral distal femoral angle
MCID: minimal clinically important difference
MP: medial‐pivot
MPTA: medial proximal tibia angle
PROMs: patient‐reported outcome measures
PS: posterior‐stabilized
PSI: patient‐specific instrumentation
ROM: range of motion
TEA: trans‐epicondylar axis
TKA: total knee arthroplasty
TMA: tibial mechanical angle
VAR: varus

## INTRODUCTION

Medial‐pivot design total knee arthroplasty (MP‐TKA) features a ball‐in‐socket design on the medial joint surface to limit motion and enhance joint stability, while the lateral side is designed to accommodate anterior‐posterior translation, thereby facilitating near‐natural knee motion during flexion [11]. Based on the National Joint Registry, MP‐TKA use is gradually increasing [23]. Several studies have reported that MP‐TKA provides superior patient‐reported outcome measures (PROMs) compared with posterior‐stabilized design TKA (PS‐TKA) [3, 18]. Since mid‐flexion instability is a known cause of dissatisfaction after TKA [29, 42], superior anteroposterior stability of MP‐TKA might contribute to these favourable outcomes [10, 39, 41, 46]. In contrast, some studies comparing PROMs between MP‐TKA and PS‐TKA have reported equivalent outcomes [6, 31]. Thus, whether MP‐TKA would provide higher patient satisfaction compared with PS‐TKA remains controversial.

Various implant manufacturers produce TKA implants, with the shape varying by manufacturer. The shape of femoral and tibial components influences post‐operative outcomes in patients undergoing TKA [24, 28, 32, 34]. Accordingly, variations in implant shape might influence outcomes when comparing PROMs in TKA across different manufacturers. However, most previous studies comparing post‐operative PROMs between MP‐TKA and PS‐TKA have used implants from different manufacturers of MP and PS implants, making a comparison of the articular surface design alone difficult [3, 6, 18, 31]. Therefore, it was hypothesized that comparing MP‐TKA and PS‐TKA with identical geometry from the same manufacturer to solely evaluate the differences in joint surface design, MP‐TKA would demonstrate better post‐operative PROMs compared with PS‐TKA, when performed under mechanical alignment. This study aimed to compare the post‐operative PROMs between MP‐TKA and PS‐TKA using implants from the same manufacturer under mechanical alignment.

## METHODS

### Study design

**Figure 1:**
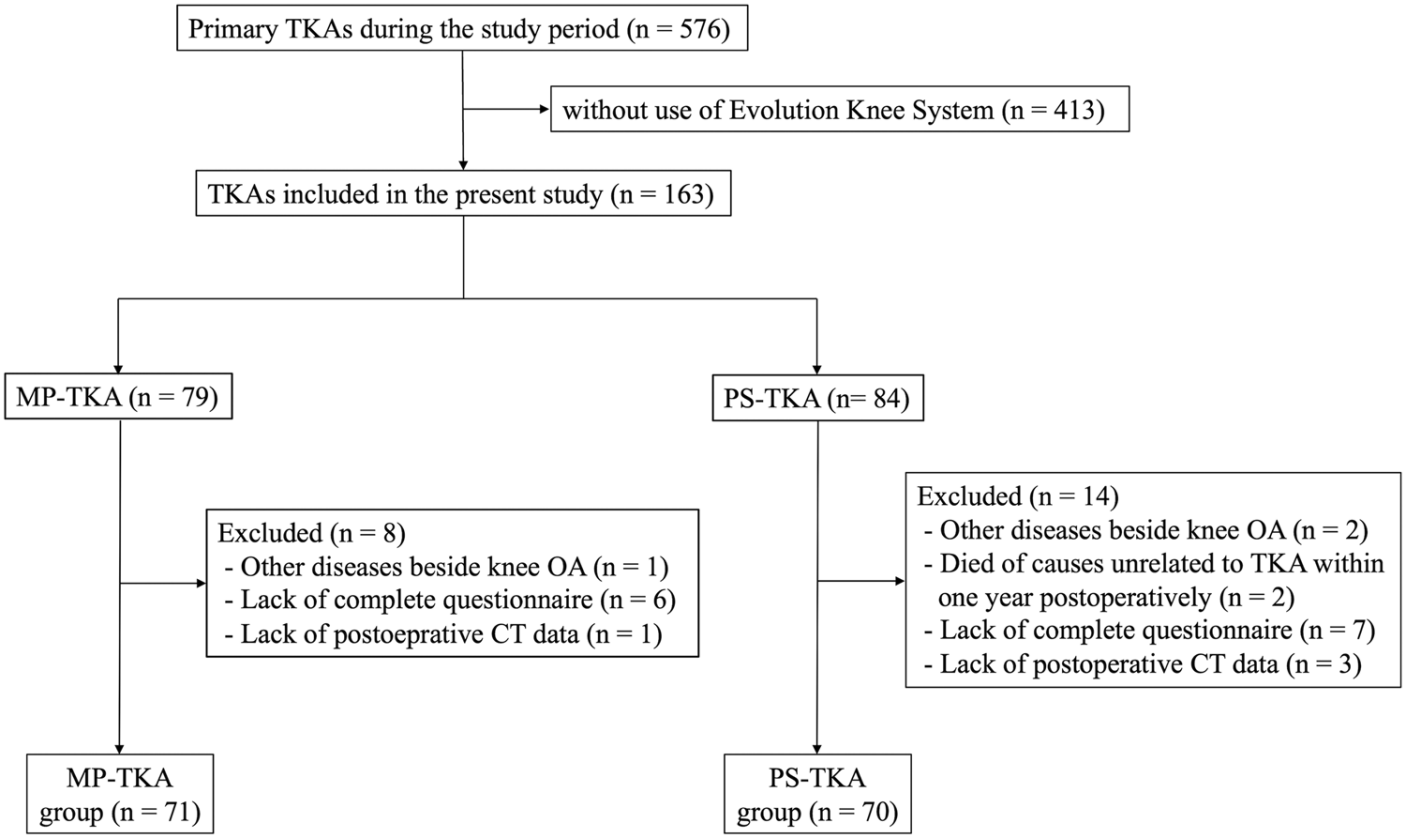
Study flow diagram. The flow diagram shows the patient recruitment process for MP‐TKA and PS‐TKA. CT, computed tomography; MP, medial‐pivot; OA, osteoarthritis; PS, posterior‐stabilized; TKA, total knee arthroplasty.

This study included 163 TKAs performed using the Evolution Knee System (MicroPort Orthopedic) out of the 576 primary TKAs performed at the institution between January 2015 and May 2021. The Evolution Knee System was used exclusively in cases of varus‐aligned knees and relatively high activity levels. To ensure a homogeneous cohort and minimize potential confounding factors, several exclusion criteria were applied. Since different underlying diseases might influence functional outcomes, knees with diagnoses other than osteoarthritis were excluded to maintain diagnostic consistency [2]. Patients with less than one year of follow‐up were excluded, because PROMs generally stabilize after this period; shorter follow‐up does not reliably reflect post‐operative outcomes [36]. Cases of incomplete questionnaire data were excluded because PROMs were considered the primary outcome measure. Knees without routine post‐operative computed tomography (CT) were excluded, because component alignment could not be evaluated, which might affect PROMs [19]. Finally, revision cases were excluded from the study to avoid potential bias from complications unrelated to implant design; however, no such cases were observed during the follow‐up period. After applying these criteria, 141 TKAs in 99 patients were included in the final analysis, comprising 71 MP‐TKAs and 70 PS‐TKAs (Figure 1).

### Implant design

**Figure 2:**
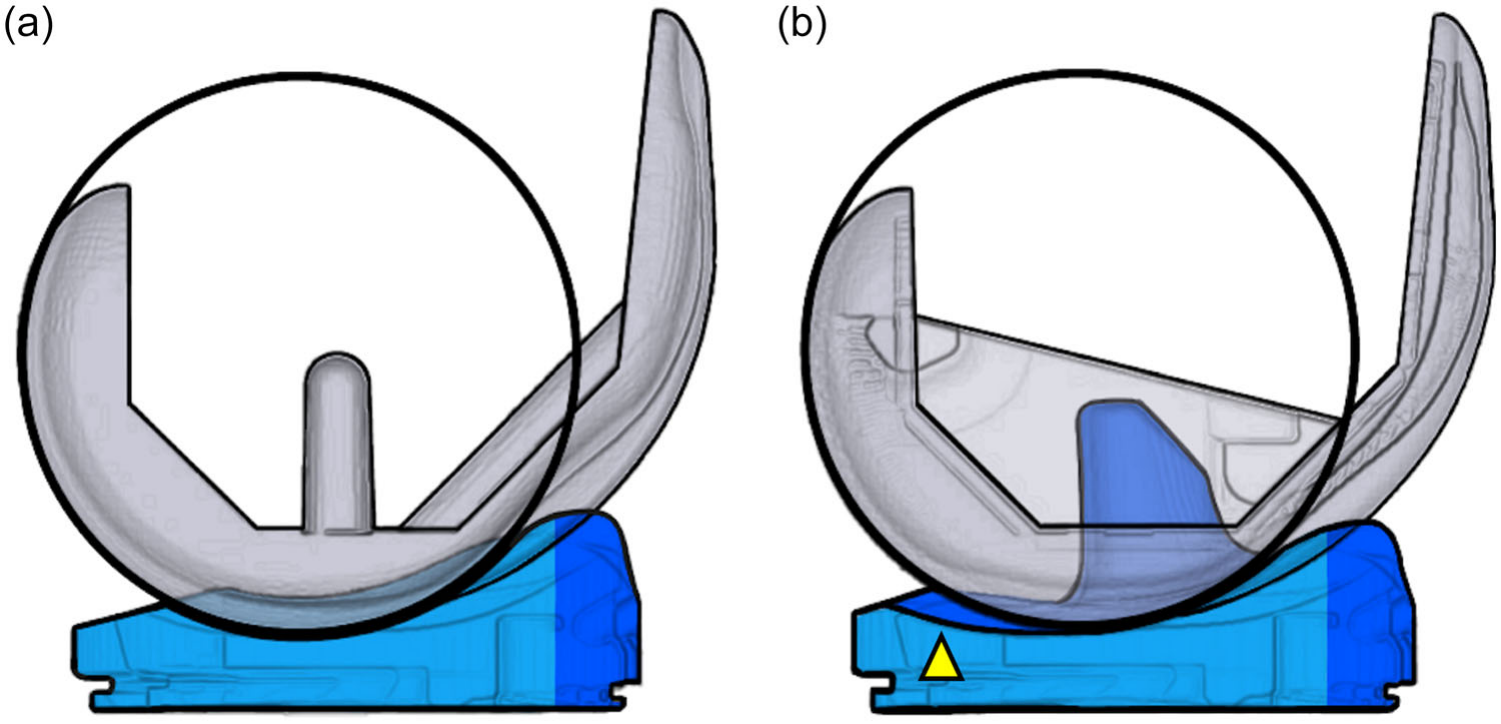
Differences in the medial joint surfaces of two types of TKA. (a) The MP design polyethylene insert features a medial joint surface configured as a complete ball‐in‐socket. (b) The PS design polyethylene insert has a medial joint surface that is not a complete ball‐in‐socket, as the posterior lip of the polyethylene insert is lowered to allow rollback through the post‐cam mechanism (arrow). MP, medial‐pivot; PS, posterior‐stabilized; TKA, total knee arthroplasty.

**Figure 3:**
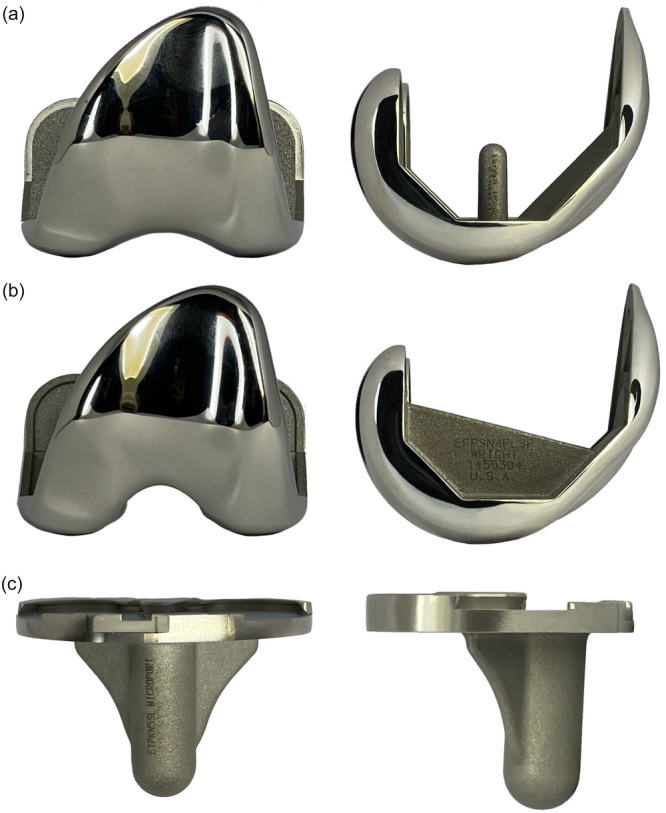
Differences in femoral and tibial components. (a) The femoral component of the MP‐TKA and (b) the femoral component of the PS‐TKA have the same shape except for the presence of the box. (c) Tibial components are identical between the two types of implants. All lateral‐view images are taken from the medial side. MP, medial‐pivot; PS, posterior‐stabilized; TKA, total knee arthroplasty.

**Figure 4:**
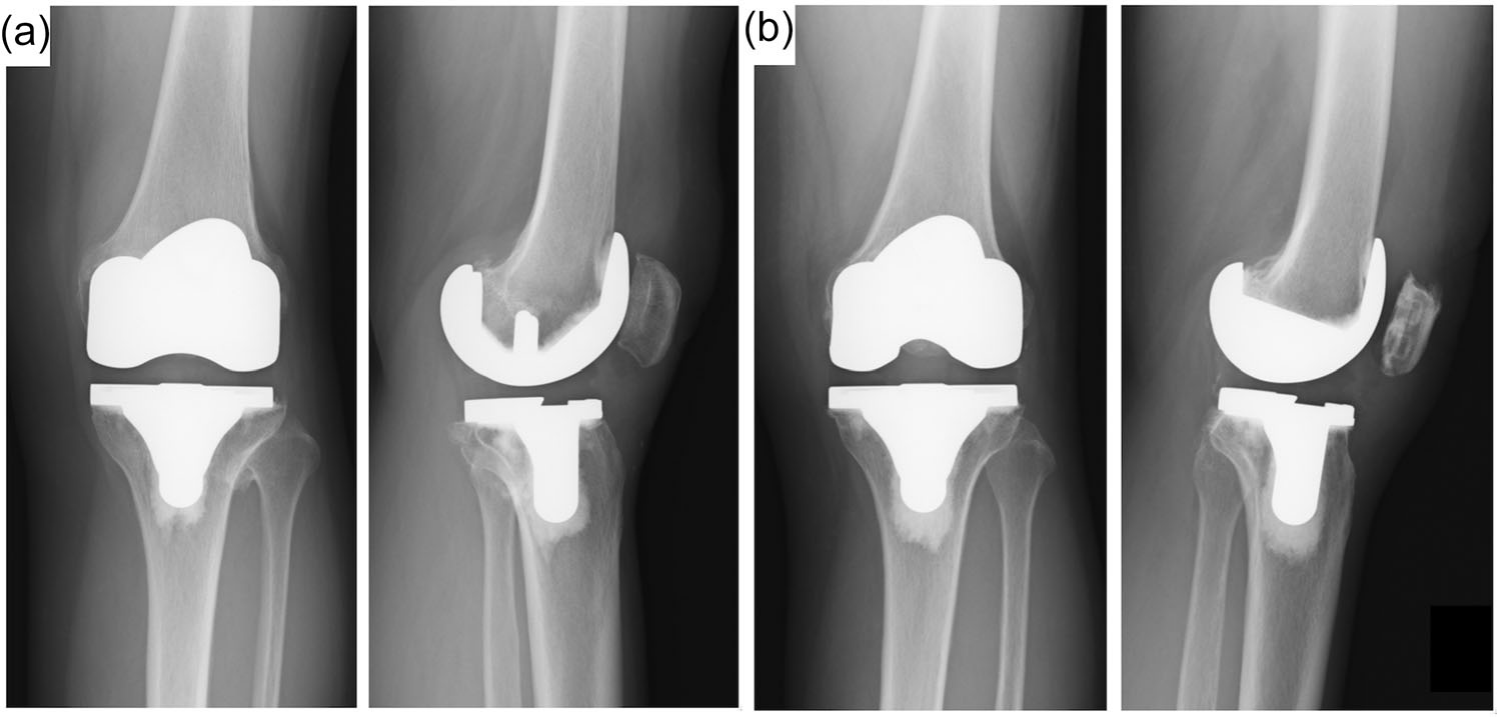
Anteroposterior and lateral plain radiographs of two types of TKA 1 year post‐operatively. (a) MP‐TKA and (b) PS‐TKA have the same shape except for the presence of the box. MP, medial‐pivot; PS, posterior‐stabilized; TKA, total knee arthroplasty.

In the MP‐TKA group, the medial joint surface of the insert forms a complete ball‐in‐socket. In contrast, in the PS‐TKA group, the posterior lip of the polyethylene insert is lowered to allow rollback through the post‐cam mechanism, meaning that the medial joint surface is not a ball‐in‐socket in this system (Figure 2). In the MP‐TKA and PS‐TKA groups, the femoral component of the implants had the same shape except for the presence of the box, and the tibial component was identical (Figure 3). A single surgical team, including four experienced arthroplasty surgeons, performed all TKAs. PS‐TKA was used at the institution before November 2017, and MP‐TKA was used thereafter. All TKA procedures were performed using a medial para‐patellar approach with a midline skin incision, and CT‐based patient‐specific instrumentation (PSI) was used in the operations [45]. The target position of the femoral and tibial components in the coronal and sagittal planes was perpendicular to the mechanical axis. In the rotational plane, the femoral component was aligned parallel to the surgical epicondylar axis; the tibial component was aligned parallel to Akagi's line [1]. Both the femoral and tibial components were cemented in all cases. In terms of patellar management, no patellar resurfacing was performed in the MP‐TKA group, whereas all knees in the PS‐TKA group underwent cemented patellar resurfacing. All patients were managed according to a standardized clinical pathway, which included the same post‐operative rehabilitation protocol, physiotherapy regimen, and discharge criteria. These protocols, including the length of hospital stay and other perioperative management parameters, remained consistent throughout the study period despite the chronological difference between the MP‐TKA and PS‐TKA groups. CT images covering the pelvis to the ankle were taken with the patient in the supine position and the knee in full extension, 2 weeks after TKA. To minimize radiation exposure, the diaphysis of the femur and tibia were excluded from the CT image range. After discharge from the hospital, patients were scheduled for regular clinical and radiological evaluations at 3, 6 and 12 months following TKA and every 1 year thereafter (Figure 4).

### Clinical outcomes

The knee range of motion (ROM) and Knee Society Score (KSS) were assessed preoperatively, and final follow‐up was conducted after TKA. Knee ROM was measured using a standard clinical goniometer based on a previously established method [14]. Post‐operative changes in knee extension and flexion angles from the preoperative values were defined as Δ knee extension and Δ knee flexion, respectively. Post‐operative complications were assessed throughout the follow‐up period, including infection, loosening, reoperations and revision surgeries.

KSS‐2011 and Forgotten Joint Score‐12 (FJS‐12) were assessed as PROMs at the final follow‐up after TKA [5, 40]. The raw FJS‐12 score was transformed to a linearly scaled score from 0 to 100, using the following formula: Final total score ＝ 100 − ([sum{item 1 to item 12} − 12]/48 × 100) [38].

### Radiological outcomes

Mechanical hip–knee–ankle angle (HKA), femoral mechanical angle (FMA), and tibial mechanical angle (TMA) were measured preoperatively and post‐operatively using standing whole‐leg radiographs [7, 12, 13, 25]. From these values, the lateral distal femoral angle (LDFA = 180 − FMA), medial proximal tibia angle (MPTA = TMA), arithmetic HKA (aHKA = MPTA − LDFA), and joint line orientation angle (= MPTA + LDFA) were calculated. These parameters were used for the coronal plane alignment of the knee (CPAK) classification [20, 27]. Alignment distribution was then classified according to both the CPAK classification and functional knee phenotypes, preoperatively and post‐operatively [13, 27].

The prosthetic alignment was measured using CT‐based three‐dimensional (3D) templating software (Zed Knee version 18.0, LEXI Co., Ltd.) based on post‐operative CT images taken 2 weeks after TKA, following a previously described method [44]. Briefly, coronal and sagittal alignments of the prosthesis were measured based on the mechanical axis defined as the line from the centre of the hip or ankle joint to the centre of the femoral and tibial components. Rotational parameters were measured based on the trans‐epicondylar axis (TEA) of the femur and Akagi's line of the tibia. The TEA was drawn from the lateral epicondyle to the most prominent point on the medial epicondyle of the femur. Akagi's line was reported to be crossing perpendicular to the TEA. It was drawn from the centre of the attachment of the posterior cruciate ligament to the medial surface of the tibial tuberosity [1]. A positive value was indicative of varus alignment of the component in the coronal plane, flexion or posterior tilt alignment of the component in the sagittal plane, and external rotation alignment of femoral component (or tibial component) relative to the clinical epicondylar axis (or Akagi's line) in the axial plane. An orthopaedic surgeon who was not involved in the surgical procedure performed the radiographic assessments.

### Institutional review board study approval

This study was conducted in accordance with the principles of the Declaration of Helsinki and was approved by the Ethics Committee of Osaka Metropolitan University (approval date: 21 February 2008; IRB No. 1280). Written informed consent was obtained from all participants prior to study initiation.

### Statistical analysis

Continuous variables are described as means with standard deviations; categorical variables are summarized using absolute frequencies and percentages. The Mann–Whitney *U* test was used for univariate analyses to assess differences in continuous variables, whereas the chi‐squared test was used for assessing differences in categorical variables between the two groups. To investigate the independent effect of the MP design prosthesis on post‐operative PROMs, multivariable linear regression analyses were conducted. In Models 1–3, the KSS‐2011 subscales (symptoms, satisfaction and functional activities) were used as dependent variables, respectively. In Model 4, the FJS‐12 total score was used as the dependent variable. In all models, the following independent variables were included: age, sex, body mass index (BMI), preoperative knee extension angle, preoperative knee flexion angle, use of the MP prosthesis, surgeon and follow‐up period. The surgeon variable was modelled using three dummy variables to represent the four surgeons, with one surgeon serving as the reference category. A power analysis was performed to determine an adequate sample size. Based on a previous study, the minimal clinically important difference (MCID) in post‐operative FJS total score was approximately 16.6 points [8]. In addition, the pilot study showed that the standard deviation of the FJS total score was 25 points. Therefore, 60 patients in each group would be needed to achieve a statistical power of 95%, with a two‐sided alpha set at 0.05. Statistical significance was set at *p* < 0.05. Statistical analyses were conducted using the R software (version 4.3.1; R Foundation for Statistical Computing).

## RESULTS

The mean follow‐up periods were 51 and 50 months in the MP‐TKA and PS‐TKA groups, respectively. Table 1 presents the patient demographics.

**Table 1:** Patient characteristics.

Parameters	MP‐TKA group (*n* = 71)	PS‐TKA group (*n* = 70)	*p*
Age at the time of operation, years	70 ± 5	70 ± 4	0.671
Woman, *n*	55 (77)	53 (76)	0.963
BMI, kg/m^2^	26.6 ± 4.9	27.4 ± 3.9	0.172
Right	37 (52)	33 (47)	0.934
ASA score, *n*			
0–2	61 (86)	61 (87)	1.000
≥3	10 (14)	9 (13)	
Follow‐up period, months	51 ± 13	50 ± 17	0.535
Clinical parameters			
Knee extension, degrees	−10 ± 8	−8 ± 7	0.423
Knee flexion, degrees	119 ± 16	126 ± 15	0.008*
KSS knee score, points	36 ± 16	38 ± 12	0.436
KSS function score, points	47 ± 17	51 ± 15	0.215

*Note*: The values are presented as mean ± standard deviations or as count (percentage).

Abbreviations: ASA, American Society of Anesthesiologists; BMI, body mass index; KSS, Knee Society Score; MP, medial‐pivot; PS, posterior‐stabilized; TKA, total knee arthroplasty.

* *p* < 0.05 was considered statistically significant.

### Clinical outcomes

The post‐operative knee flexion angle was smaller in the MP‐TKA group than in the PS‐TKA group at final follow‐up after TKA (124 ± 13 vs. 132 ± 13, *p* < 0.001); however, Δ knee flexion angle did not differ between the two groups (5 ± 15 vs. 6 ± 15, *p* = 0.752). KSS function score was better in the MP‐TKA group than in the PS‐TKA group (92 ± 10 vs. 87 ± 11, *p* = 0.015) (Table 2). No complications, reoperations or revision surgeries were observed in either group during the follow‐up period.

**Table 2:** Clinical outcomes at the final follow‐up after TKA.

Parameters	MP‐TKA group (*n* = 71)	PS‐TKA group (*n* = 70)	*p*
Knee extension, degrees	−2 ± 2	−5 ± 2	0.774
Knee flexion, degrees	124 ± 13	132 ± 13	<0.001*
ΔKnee extension, degrees	9 ± 8	7 ± 7	0.224
ΔKnee Flexion, degrees	5 ± 15	6 ± 15	0.752
KSS knee score, points	96 ± 5	97 ± 4	0.894
KSS function score, points	92 ± 10	87 ± 11	0.015*

*Note*: The values are presented as mean ± standard deviations.

Abbreviations: KSS, Knee Society Score; MP, medial‐pivot; PS, posterior‐stabilized; TKA, total knee arthroplasty.

* *p* < 0.05 was considered statistically significant.

KSS‐2011 scores for symptoms (22 ± 3 vs. 19 ± 6, *p* = 0.002), satisfaction (32 ± 8 vs. 28 ± 10, *p* = 0.020) and functional activities (79 ± 16 vs. 71 ± 22, *p* = 0.041), as well as FJS‐12 total scores (73 ± 24 vs. 64 ± 27, *p* = 0.025), were higher in the MP‐TKA group than in the PS‐TKA group. Several FJS sub‐items also showed better results in the MP‐TKA group (Table 3).

**Table 3:** PROMs at the final follow‐up after TKA.

Parameters	MP‐TKA group (*n* = 71)	PS‐TKA group (*n* = 70)	*p*
KSS‐2011, points			
Symptoms	22 ± 3	19 ± 6	0.002*
Satisfaction	32 ± 8	28 ± 10	0.020*
Expectation	10 ± 2	10 ± 2	0.817
Functional activities	79 ± 16	71 ± 22	0.041*
FJS‐12, points			
Total score	73 ± 24	64 ± 27	0.025*
Q1. In bed at night	0.6 ± 0.9	1.1 ± 1.2	0.006*
Q2. Sitting on a chair for more than 1 h	0.8 ± 1.1	1.3 ± 1.2	0.022*
Q3. Walking for more than 15 min	0.8 ± 1.1	1.2 ± 1.4	0.146
Q4. Taking a bath/shower	0.6 ± 0.9	1.0 ± 1.2	0.125
Q5. Travelling in a car	0.8 ± 1.0	1.0 ± 1.2	0.332
Q6. Climbing stairs	1.4 ± 1.3	1.7 ± 1.4	0.171
Q7. Walking on uneven ground	1.3 ± 1.2	1.9 ± 1.4	0.006*
Q8. Standing up from a low‐sitting position	1.3 ± 1.2	1.9 ± 1.3	0.012*
Q9. Standing for long periods of time	1.3 ± 1.1	1.7 ± 1.2	0.043*
Q10. Doing housework or gardening	1.2 ± 1.1	1.6 ± 1.3	0.135
Q11. Taking a walk/hiking	1.3 ± 1.2	1.7 ± 1.3	0.087
Q12. Doing your favourite sport	1.4 ± 1.2	1.5 ± 1.3	0.499

*Note*: The values are presented as mean ± standard deviations.

Abbreviations: FJS, Forgotten Joint Score; KSS, Knee Society Score; MP, medial‐pivot; PROMs, patient‐reported outcome measures; PS, posterior‐stabilized; TKA, total knee arthroplasty.

* *p* < 0.05 was considered statistically significant.

Using multivariable analysis, the use of an MP design prosthesis was independently associated with higher PROMs, including symptoms, satisfaction, and functional activities (Table 4).

**Table 4:** Multiple linear regression analysis to identify factors associated with PROMs.

Predictors	Partial regression coefficient (95% CI)	*p*
Model 1: KSS‐2011 symptoms		
Age at operation, years	0.01 (−0.2 to 0.2)	0.895
Sex (Men)	−0.5 (−2.3 to 1.3)	0.588
BMI, kg/m^2^	−0.2 (−0.3 to 0.03)	0.091
Preoperative knee extension, degrees	−0.01 (−0.1 to 0.1)	0.819
Preoperative knee flexion, degrees	0.01 (−0.04 to 0.06)	0.763
Medial‐pivot design prosthesis	2.8 (1.3–4.4)	<0.001*
Surgeons (ref: Surgeon A)		
Surgeon B	1.6 (−2.5 to 5.7)	0.444
Surgeon C	0.4 (−2.1 to 3.0)	0.735
Surgeon D	−0.9 (−3.8 to 2.0)	0.538
Follow‐up period, months	0.1 (0.1–0.2)	<0.001*
Model 2: KSS‐2011 satisfaction		
Age at operation, years	−0.2 (−0.5 to 0.1)	0.226
Sex (Men)	0.6 (−3.0 to 4.1)	0.743
BMI, kg/m^2^	−0.1 (−0.5 to 0.2)	0.465
Preoperative knee extension, degrees	−0.04 (−0.3 to 0.2)	0.691
Preoperative knee flexion, degrees	0.02 (−0.1 to 0.1)	0.639
Medial‐pivot design prosthesis	3.9 (0.9–7.0)	0.013*
Surgeons (ref: Surgeon A)		
Surgeon B	2.7 (−5.5 to 10.8)	0.518
Surgeon C	−1.0 (−6.1 to 4.0)	0.693
Surgeon D	−0.2 (−6.0 to 5.5)	0.933
Follow‐up period, months	0.1 (0.04–0.2)	0.005*
Model 3: KSS‐2011 functional activities		
Age at operation, years	−0.6 (−1.3 to 0.1)	0.106
Sex (Men)	2.5 (−4.9 to 9.8)	0.510
BMI, kg/m^2^	−1.2 (−1.9 to −0.4)	0.003*
Preoperative knee extension, degrees	−0.3 (−0.7 to 0.1)	0.183
Preoperative knee flexion, degrees	0.04 (−0.2 to 0.3)	0.720
Medial‐pivot design prosthesis	6.6 (0.2–13.0)	0.043*
Surgeons (ref: Surgeon A)		
Surgeon B	−1.2 (−18.2 to 15.7)	0.886
Surgeon C	−1.5 (−12.0 to 9.0)	0.781
Surgeon D	−2.4 (−14.4 to 9.5)	0.686
Follow‐up period, months	0.4 (0.2–0.6)	<0.001*
Model 4: FJS‐12 total score		
Age at operation, years	−0.3 (−1.3 to 0.7)	0.582
Sex (Men)	−3.1 (−13.5 to 7.3)	0.559
BMI, kg/m^2^	−0.2 (−1.2 to 0.9)	0.783
Preoperative knee extension, degrees	−0.01 (−0.6 to 0.6)	0.977
Preoperative knee flexion, degrees	−0.1 (−0.4 to 0.2)	0.434
Medial‐pivot design prosthesis	9.0 (−0.1 to 18.2)	0.054
Surgeons (ref: Surgeon A)		
Surgeon B	3.1 (−20.6 to 26.8)	0.795
Surgeon C	−4.2 (−19.3 to 11.0)	0.587
Surgeon D	0.6 (−16.1 to 17.3)	0.945
Follow‐up period, months	0.3 (0.03–0.6)	0.029*

*Note*: Surgeon A was used as the reference category for the dummy variables of the surgeon variable.

Abbreviations: BMI, body mass index; CI, confidence interval; FJS, Forgotten Joint Score; KSS, Knee Society Score; PROMs, patient‐reported outcome measures.

* *p* < 0.05 was considered statistically significant.

### Radiological outcomes

**Figure 5:**
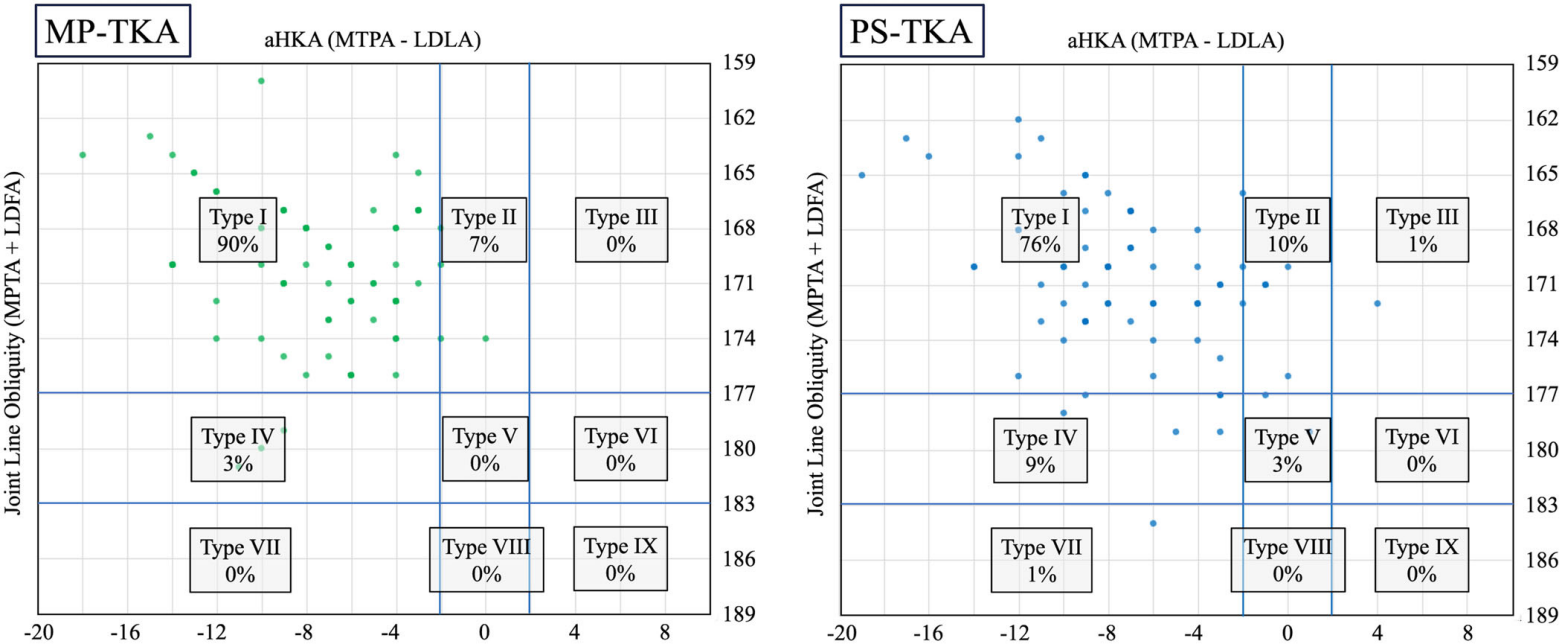
Distribution of preoperative CPAK classification. The distribution of preoperative CPAK classification is shown for each group: the MP‐TKA group on the left and the PS‐TKA group on the right. aHKA, arithmetic hip−knee–ankle angle; CPAK, coronal plane alignment of the knee; LDFA, lateral distal femoral angle; MP, medial‐pivot; MPTA, medial proximal tibia angle; PS, posterior‐stabilized; TKA, total knee arthroplasty.

**Figure 6:**
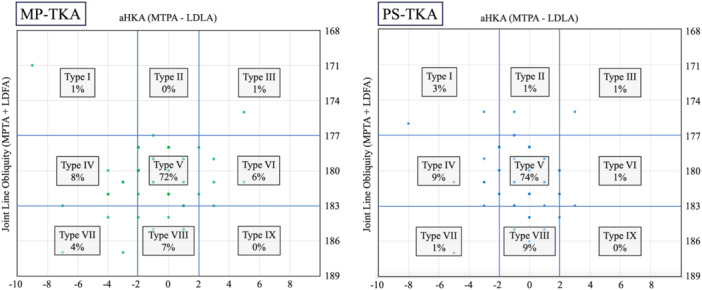
Distribution of post‐operative CPAK classification. The distribution of post‐operative CPAK classification is shown for each group: the MP‐TKA group on the left and the PS‐TKA group on the right. aHKA, arithmetic hip–knee–ankle angle; CPAK, coronal plane alignment of the knee; LDFA, lateral distal femoral angle; MP, medial‐pivot; MPTA, medial proximal tibia angle; PS, posterior‐stabilized; TKA, total knee arthroplasty.

Pre‐ and post‐operative parameters are presented in Table 5. In the CPAK classification, type I was the most common preoperatively in both the MP‐TKA and PS‐TKA groups (Figure 5), whereas type V was the most common post‐operatively (Figure 6). Regarding functional knee phenotypes, both the MP‐TKA and PS‐TKA groups most frequently exhibited varus (VAR) HKA 9° VAR FMA 3° VAR TMA 3° preoperatively, whereas neutral HKA 0° VAR FMA 3° valgus TMA 3° was the most common phenotype post‐operatively (Table 6).

**Table 5:** Radiographic outcomes assessed by standing whole‐leg radiographs.

Parameters	MP‐TKA group (*n* = 71)	PS‐TKA group (*n* = 70)	*p*
Preoperative parameters			
mHKA	167 ± 6	167 ± 7	0.603
aHKA	−8 ± 4	−7 ± 5	0.777
FMA	91 ± 2	91 ± 2	0.733
TMA (MPTA)	81 ± 3	82 ± 4	0.606
LDFA	89 ± 2	89 ± 2	0.733
JLO	170 ± 4	171 ± 5	0.510
Post‐operative parameters			
mHKA	181 ± 2	181 ± 3	0.225
aHKA	−1 ± 2	−1 ± 2	0.314
FMA	89 ± 2	90 ± 2	0.342
TMA (MPTA)	90 ± 2	90 ± 2	0.960
LDFA	91 ± 2	90 ± 2	0.342
JLO	181 ± 3	180 ± 3	0.470

*Note*: The values are presented as mean ± standard deviations.

Abbreviations: aHKA, arithmetic hip–knee–ankle angle; FMA, femoral mechanical angle; JLO, joint line orientation angle; LDFA, lateral distal femoral angle; mHKA, mechanical hip–knee–ankle angle; MPTA, medial proximal tibia angle; MP, medial‐pivot; PS, posterior‐stabilized; TKA, total knee arthroplasty; TMA, tibial mechanical angle.

**Table 6:** Pre‐ and post‐operative functional knee phenotypes.

Rank	MP‐TKA group (*n* = 71)		PS‐TKA group (*n* = 70)	
	*n* (%)	Functional knee phenotypes	*n* (%)	Functional knee phenotypes
Preoperative				
1	10 (14)	VAR HKA9° VAR FMA3° VAR TMA3°	7 (10)	VAR HKA9° VAR FMA3° VAR TMA3°
2	5 (7)	VAR HKA6° VAR FMA3° NEU TMA0°	6 (9)	VAR HKA12° VAR FMA3° VAR TMA3°
3	5 (7)	VAR HKA6° NEU FMA0° VAR TMA3°	5 (7)	VAR HKA9° VAR FMA3° NEU TMA0°
Total	20 (28)		18 (26)	
Post‐operative				
1	59 (83)	NEU HKA0° VAR FMA3° VAL TMA3°	61 (87)	NEU HKA0° VAR FMA3° VAL TMA3°
2	5 (7)	VAR HKA3° VAR FMA3° VAL TMA3°	5 (7)	VAR HKA3° VAR FMA3° VAL TMA3°
Total	64 (90)		66 (94)	

*Note*: The values are presented as count (percentage).

Abbreviations: FMA, femoral mechanical angle; HKA, hip–knee–ankle angle; MP, medial‐pivot; NEU, neutral; PS, posterior‐stabilized; TKA, total knee arthroplasty; TMA, tibial mechanical angle; VAL, valgus; VAR, varus.

3D prosthetic alignment measured on CT did not differ significantly between the two groups (Table 7).

**Table 7:** Three‐dimensional prosthetic alignment assessed using CT.

Parameters	MP‐TKA group (*n* = 71)	PS‐TKA group (*n* = 70)	*p*
Coronal parameters, degrees			
Femoral component alignment	1.2 ± 2.2	0.8 ± 1.7	0.474
Tibial component alignment	0.2 ± 1.4	0.3 ± 1.6	0.918
Sagittal parameters, degrees			
Femoral component alignment	1.4 ± 3.0	0.8 ± 2.9	0.861
Tibial component alignment	2.9 ± 2.6	2.4 ± 2.3	0.160
Rotational parameters, degrees			
Femoral component alignment	−2.7 ± 3.1	−3.0 ± 2.5	0.588
Tibial component alignment	1.7 ± 4.3	1.2 ± 5.0	0.441

*Note*: The values are presented as mean ± standard deviations.

Abbreviations: CT, computed tomography; MP, medial‐pivot; PS, posterior‐stabilized; TKA, total knee arthroplasty.

## DISCUSSION

This study demonstrated that the MP‐TKA group resulted in better post‐operative KSS‐2011 and FJS‐12 scores than did the PS‐TKA group under mechanical alignment conditions. A key strength of this study is that it compared MP‐TKA and PS‐TKA using implants from the same manufacturer, ensuring identical shape. Therefore, the comparison of PROMs solely reflects the differences in articular surface design between MP‐TKA and PS‐TKA.

Compared with the PS‐TKA group, the MP‐TKA group showed better outcomes in KSS‐2011 scores for symptoms, satisfaction, and functional activities. Furthermore, the differences in KSS‐2011 scores between MP‐TKA and PS‐TKA met the previously reported MCID [33]. MP‐TKA provides better antero‐posterior stability compared with PS‐TKA [10, 39, 41, 46], resulting in reduced post‐operative instability. Since knee instability following TKA is associated with post‐operative PROMs [10, 43], these findings suggest that differences in articular surface design associated with stability in TKA might directly affect post‐operative PROMs. In addition, this study was conducted exclusively under mechanical alignment. However, a previous study has reported that PROMs of MP‐TKA are more favourable when performed with kinematic alignment compared with mechanical alignment [9]. Moreover, MP‐TKA has been shown to demonstrate greater medial pivot motion under kinematic alignment than under mechanical alignment [4, 17]. These findings suggest that performing MP‐TKA with kinematic alignment might yield even greater improvements in patient satisfaction. Further investigations are needed to validate this possibility.

The FJS‐12 total score was higher in the MP‐TKA group than in the PS‐TKA group. Compared with PS‐TKA, MP‐TKA showed superior outcomes for walking on uneven ground and standing up from a low position, likely owing to its superior antero‐posterior stability [10, 39, 41, 46]. However, caution is needed when interpreting these results. Using the multivariable analysis, the use of the MP design prosthesis was not an independent predictor of the FJS‐12 total score, suggesting that the observed difference may have been influenced by confounding factors. Furthermore, the difference in the FJS‐12 total score between MP‐TKA and PS‐TKA in this study did not exceed the previously reported MCID [8]. Whether the FJS difference observed in this study would have clinical relevance remains unclear, highlighting the need for further research.

This study showed that a greater post‐operative flexion angle was observed in the PS‐TKA group compared with the MP‐TKA group. The greater flexion angle in PS‐TKA might be attributed to the rollback induced via the post‐cam mechanism during knee flexion. However, this study showed no difference in the Δ knee flexion angle between MP‐TKA and PS‐TKA. The preoperative ROM influences post‐operative ROM after TKA [22]. Some studies comparing MP‐TKA and PS‐TKA have reported no differences in ROM [16, 26]. Therefore, the better post‐operative flexion angle observed in the PS‐TKA group in this study might be attributed to the superior preoperative flexion angle in that group.

A major strength of this study was that it was the first to compare PROMs between MP‐TKA and PS‐TKA using the Evolution Knee System. Even among PS‐TKA designs, post‐operative outcomes can vary depending on the manufacturer [15]. In this study, such implant design variability was eliminated using components from the same manufacturer. Additionally, there were no significant differences in post‐operative 3D‐measured alignment between the two groups. Given that post‐operative component alignment has been associated with post‐operative PROMs [19], controlling for this variable further strengthens the validity of this study. The use of PSI allowed for precise osteotomy, thereby minimizing alignment bias between the groups.

This study had several limitations. First, as it only included patients with osteoarthritis, the results are not generalizable to patients with other knee pathologies. Further studies are required to address this issue. Second, the patellar resurfacing status differed between the two groups. This surgical decision was based on the high revision rates associated with non‐resurfaced PS‐TKA, as reported by the National Joint Registry [23]. Despite lacking patellar resurfacing in the MP‐TKA group, no patients experienced anterior knee pain or required secondary patellar resurfacing. Therefore, the impact of differences in patellar management on the clinical outcomes in this study is likely to have been minimal. Third, the average BMI of patients in both groups was relatively low compared with TKA populations in Western countries. According to national joint registries from the United States, Australia and the United Kingdom (specifically, England, Wales and Northern Ireland), more than half of patients with TKA in those regions are classified as obese (BMI ≥ 30) [21, 23, 35]. In contrast, the mean BMI in this cohort was 26.6 kg/m^2^ in the MP‐TKA group and 27.4 kg/m^2^ in the PS‐TKA group. Although these values might appear low by Western standards, they are representative of typical TKA populations in Asian countries [30, 37]. Nevertheless, the results of this study might not be directly generalizable to populations with higher BMI. Further research is warranted to evaluate the clinical performance and durability of these prostheses in patients with obesity. Fourth, only patients with varus knees were included in this study. Therefore, the results might not be generalizable to patients with valgus knees, who might require different alignment strategies or exhibit different clinical outcomes. Further studies are warranted to evaluate the applicability of these findings in patients with valgus deformity. Fifth, the Evolution Knee System was not used in all cases during the study period, which might have introduced selection bias. However, there were no significant differences in baseline patient characteristics between the MP‐TKA and PS‐TKA groups, except for the preoperative flexion angle. Therefore, this potential bias is unlikely to have affected the primary aim of comparing MP‐TKA and PS‐TKA. Nonetheless, since the outcomes might vary in different cohorts from the current inclusion criteria, further studies are needed to investigate these findings.

Despite these limitations, the present results provided important insights into improving clinical practice. MP‐TKA resulted in higher post‐operative satisfaction than did PS‐TKA under mechanical alignment, even when the influence of implant geometry was eliminated. This finding highlights the clinical relevance that MP‐TKA should be considered when maximizing patient satisfaction is a priority.

## CONCLUSIONS

When solely comparing articular surface design and excluding variation in implant shape, MP‐TKA demonstrated better post‐operative PROMs than did PS‐TKA under mechanical alignment. Implant design with greater antero‐posterior stability could improve post‐operative outcomes after TKA.

## AUTHOR CONTRIBUTIONS

All authors contributed to the study conception and design. Material preparation, data collection and investigation were performed by Sho Masuda, Hideki Ueyama, Yohei Ohyama and Ryo Sugama. Formal analysis and data visualization were performed by Sho Masuda. Project administration and overall supervision were conducted by Yukihide Minoda and Hidetomi Terai. The first draft of the manuscript was written by Sho Masuda, and all authors contributed to the review and editing of the manuscript. All authors read and approved the final manuscript.

## CONFLICT OF INTEREST STATEMENT

The authors declare no conflicts of interest.

## ETHICS STATEMENT

This study was performed in line with the principles of the Declaration of Helsinki. Approval was granted by the Ethics Committee of Osaka Metropolitan University Graduate School of Medicine (approval no. 1280) on 21 February 2008. Informed consent was obtained from all individual participants included in the study.

## Data Availability

The data that support the findings of this study are available from the corresponding author upon reasonable request.
